# Supplementary material for: A Global Screen for Assembly State Changes of the Mitotic Proteome by SEC-SWATH-MS
Source: Cell Syst. 2020 Feb 26;10(2):133–155.e6. doi: 10.1016/j.cels.2020.01.001 (PMC7042714; doi:10.1016/j.cels.2020.01.001)

Q9Y5L4 | TIM13_HUMAN | TIMM13 TIM13B TIMM13A TIMM13B
Monomer MW [kDa]: 10.5 Monomer expected elution fraction: 60

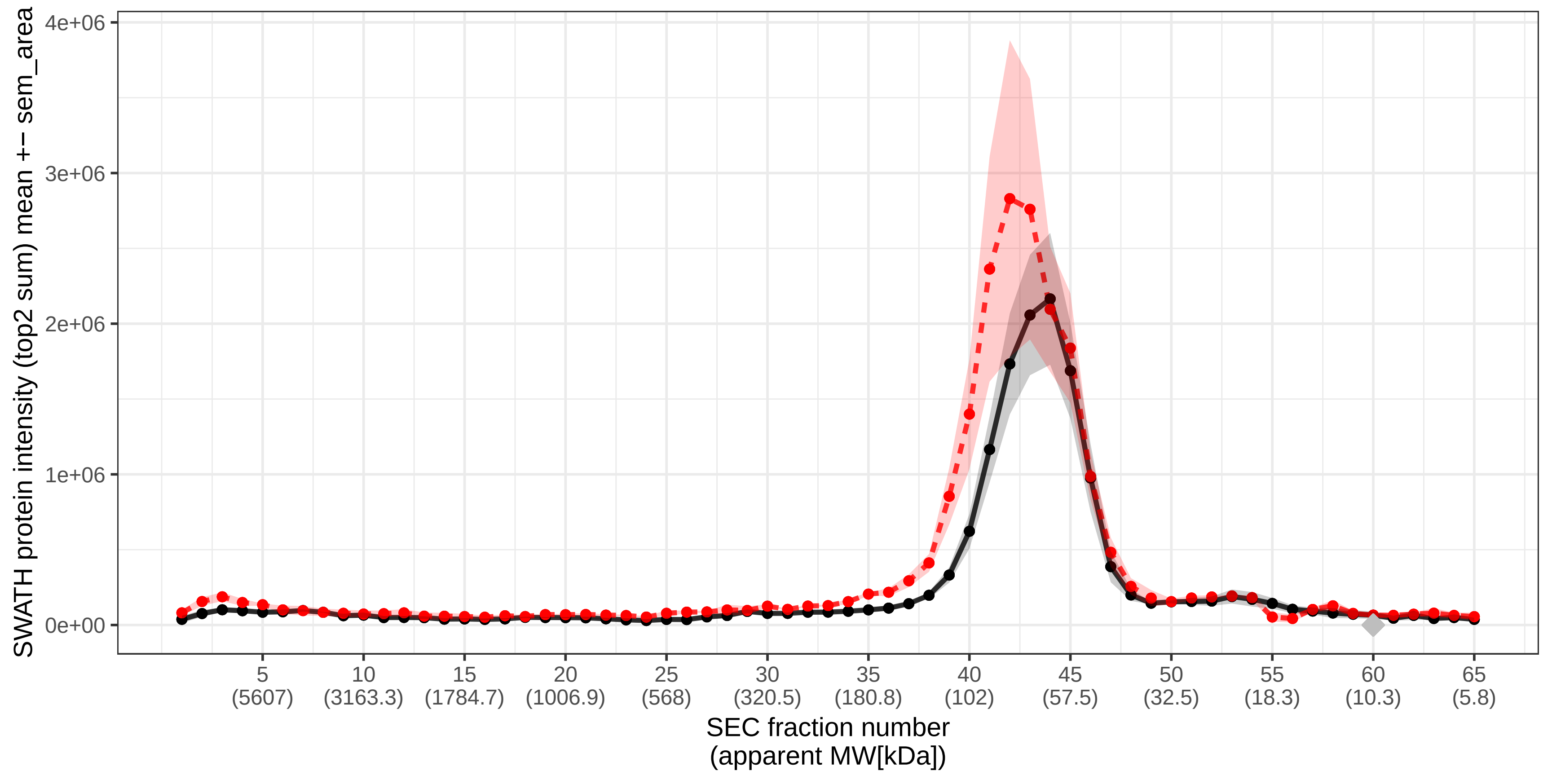

Supplement: Data S1. SEC-SWATH-MS Protein Chromatograms, Related to Figure 1 [file mmc6.zip › SECchrom_Q9Y5L4_TIM13_HUMAN_TIMM13_TIM13B_TIMM13A_TIMM13B.pdf]
